# Aberrant NF-κB activation in odontoblasts orchestrates inflammatory matrix degradation and mineral resorption

**DOI:** 10.1038/s41368-022-00159-3

**Published:** 2022-01-26

**Authors:** Fanyuan Yu, Fengli Huo, Feifei Li, Yanqin Zuo, Chenglin Wang, Ling Ye

**Affiliations:** 1 State Key Laboratory of Oral Diseases & National Clinical Research Center for Oral Diseases, West China Hospital of Stomatology, Sichuan University, Chengdu, China; 2 Department of Endodontics, West China Stomatology Hospital, Sichuan University, Chengdu, China

**Keywords:** Pulpitis, Proteases

## Abstract

Inflammation-associated proteinase functions are key determinants of inflammatory stromal tissues deconstruction. As a specialized inflammatory pathological process, dental internal resorption (IR) includes both soft and hard tissues deconstruction within the dentin-pulp complex, which has been one of the main reasons for inflammatory tooth loss. Mechanisms of inflammatory matrix degradation and tissue resorption in IR are largely unclear. In this study, we used a combination of Cre-loxP reporter, flow cytometry, cell transplantation, and enzyme activities assay to mechanistically investigate the role of regenerative cells, odontoblasts (ODs), in inflammatory mineral resorption and matrices degradation. We report that inflamed ODs have strong capabilities of matrix degradation and tissue resorption. Traditionally, ODs are regarded as hard-tissue regenerative cells; however, our data unexpectedly present ODs as a crucial population that participates in IR-associated tissue deconstruction. Specifically, we uncovered that nuclear factor-kappa b (NF-κB) signaling orchestrated Tumor necrosis factor α (TNF-α)-induced matrix metalloproteinases (Mmps) and Cathepsin K (Ctsk) functions in ODs to enhance matrix degradation and tissue resorption. Furthermore, TNF-α increases Rankl/Opg ratio in ODs via NF-κB signaling by impairing Opg expression but increasing Rankl level, which utterly makes ODs cell line 17IIA11 (A11) become Trap^+^ and Ctsk^+^ multinucleated cells to perform resorptive actions. Blocking of NF-κB signaling significantly rescues matrix degradation and resorptive functions of inflamed ODs via repressing vital inflammatory proteinases Mmps and Ctsk. Utterly, via utilizing NF-κB specific small molecule inhibitors we satisfactorily attenuated inflammatory ODs-associated human dental IR in vivo. Our data reveal the underlying mechanisms of inflammatory matrix degradation and resorption via proteinase activities in IR-related pathological conditions.

## Introduction

Dental internal resorption (IR) begins in the dental-pulpal space and extends into surrounding dentin, which includes both dental pulp matrix degradation and dentin resorption.^1,2^ IR is a typically pathological resorption, which is usually initiated via continuous inflammation in the pulp.^1^ Although some studies indicate that osteoclasts (OCs), or odontoclasts (ONCs) could be the specific cell populations that perform tissue deconstructions in IR, there remains largely unanswered as the sources of ONCs, differences between OCs and ONCs, direct clinical evidences of ONCs, and so on.^1–3^ Overall, the underlying mechanisms of deconstructive functions in IR are yet unclear. Except from OCs and ONCs, odontoblasts (ODs) are unexpectedly hypothesized to play a role in IR.^4–6^ As a specialized population in dentin-pulp complex, ODs are traditionally regarded as hard-tissue regenerative cells.^7^ However, more and more researches have shown that ODs probably also owned catabolic capabilities.^4,5,8–11^

Matrix metalloproteinases (Mmps) and Cathepsin K (Ctsk) constitute a compensatory but parallel network to orchestrate OCs-mediated bone resorption.^12^ But with respect to the roles of Mmps and Ctsk in pulp-dentin remodeling or inflammatory resorption, evidences and mechanisms are far unclear. Mmps (member 1, 2, 9, 10, 11, 13, 14, 15, 16, 17, 19, 20, and 23) are commonly expressed in both ODs and pulp cells.^13^ Ctsk can be significantly induced via Rankl in ODs-like MDPC-23 cells.^6^ In details, after the treatment of Rankl and Macrophage colony-stimulating factor (Mcsf), MDPC-23 cell formed Trap^+^ multinucleated giant cells, which demonstrated the abilities of dentin resorption in vitro.^6^ Under inflammatory conditions, Mmps, especially Mmp1, 2, 9, and 13 are significantly upregulated in the pulp-dentin complex.^14–21^ These findings indicate that under inflammation conditions, matrix degradation and dentin resorption can be assumed to be partially achieved via ODs-derived proteases, including Mmps and Ctsk.^17,18,22,23^ Despite these important findings, however, mechanisms of how inflammatory factors increase Mmps and Ctsk expressions in ODs are totally unclear.

ODs and dental pulp cells are two major but distinct cell populations within the dentin-pulp complex. A research on dental pulp cells showed that at the early phase of pulp inflammation tumor necrosis factor-α (TNF-α) induced pulp cells differentiation into ODs, but at this stage there were very rare expressions of Mmps in inflamed-dental pulp cells.^24^ It implies that under inflammatory conditions, pulp cells preferentially exhibit protect functions to form dentin rather than to degrade or resorb tissues.^24^ It is intriguing that both proteomic and transcriptomic analysis demonstrated that there existed remarkable distinctions between ODs and pulp cells.^25,26^ Besides, under the stimulations of different oral pathogens, ODs and pulp cells also presented different expressions of chemokines and inflammatory cytokines.^11^ These clues imply that ODs may play diverse roles in tissue deconstruction and immunity regulations in comparison to pulp cells. However, it is unclear whether ODs can demonstrate destructive features in IR conditions.

Here, in this study we aim to investigate the roles of ODs in inflammation-related matrix degradation and tissue resorption. Our results depict crucial roles of ODs in inflammatory matrix degradation and tissue resorption, and demonstrate the vital functions of NF-κB on orchestrating ODs-dependent destructive capabilities. These data unexpectedly present certain possible roles of ODs in IR-associated tissue deconstruction.

## Results

### Mmps expression in inflamed ODs in vivo and in vitro

**Fig. 1:**
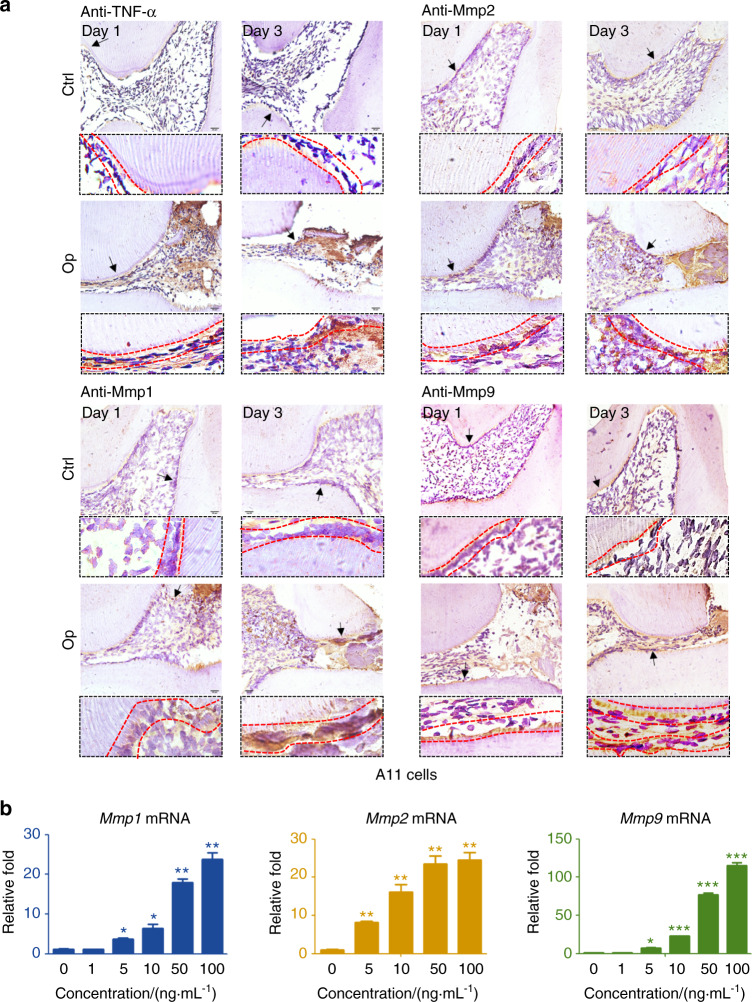
In vivo and in vitro measurements of Mmps in inflamed ODs. **a** IHC staining of TNF-α and vital Mmps in inflamed ODs. Black arrows indicated the locations of high magnification images. ODs layers were contoured using red dot lines. Counterstained using hematoxylin. The cell layers within the red-dotted lines were OD cells via their obvious and typical anatomic characteristics, such as the closest cells adjacent to dentine and so on. **b** RT-qPCR data of Mmps at 24 h post-treatments of TNF-α in A11. The concentrations listed indicated the doses of TNF-α. A two-tailed Student’s *t* test was used to determine the significance of difference as 0 ng·mL^−1^ TNF-α was set as control. *P < 0.05; **P < 0.01; ***P < 0.001; ns, no statistical significance

To detect Mmps in inflamed ODs we conducted pulp infection model (Figs. 1a and S1A). IHC data clearly showed that 1 d after surgery the level of TNF-α in ODs was significantly upregulated, and the expression of TNF-α was continuously increased at 3 d postsurgery (Fig. 1a). These IHC data of TNF-α, in combination with H&E results (Figs. S1A and B), demonstrated the remarkably strengthened inflammation in ODs after experimental pulp infection. Thus, we further detected Mmp1, 2, and 9 levels in these inflamed ODs. Data showed that the expressions of Mmp1, 2, 9, and 13 in ODs were rapidly and obviously upregulated both at 1 d and 3 d after surgery (Figs. 1a and S2, S3). Taken the findings that TNF-α level in inflamed ODs was significantly upregulated both at 1 d and 3 d postsurgery (Fig. 1a), we further detected the expressions of Mmps under TNF-α stimulation in vitro. Data showed that Mmp1, 2, 9, and 13 were all induced by TNF-α in both ODs cell line 17IIA11 (A11)^27^ and primary OD cells, and the expressions of these Mmps rose along with the increment of TNF-α concentrations (Figs. 1b and S1C, S1D).

### In vivo and in vitro Ctsk expressions in inflamed ODs

**Fig. 2:**
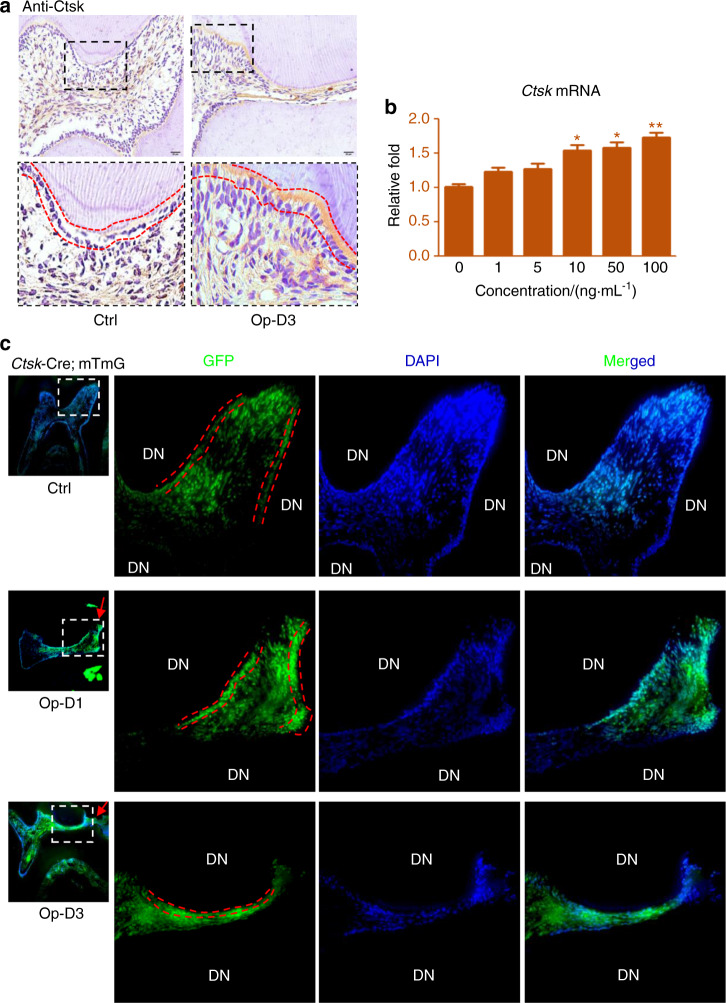
In vivo and in vitro measurements of Ctsk in inflamed ODs. **a** IHC staining of Ctsk in inflamed ODs. Boxed areas indicated the locations of high magnification images. ODs layers were contoured using red-dotted lines. Counterstained using hematoxylin. Op-D3, 3 d postsurgery. **b** RT-qPCR data of Ctsk at 24 h post-treatments of TNF-α in A11. The concentrations listed indicated the doses of TNF-α. A two-tailed Student’s *t* test was used to determine the significance of difference as 0 ng·mL^−1^ TNF-α was set as control. *P < 0.05; **P < 0.01; ns, no statistical significance. **c** Immunofluorescence images of Ctsk-Cre; mTmG mice which were subjected to experimental pulp infection surgeries. DN, dentin; Boxed areas indicated the locations of high magnification images. ODs layers were contoured using red dot lines. Counterstained using DAPI. Op-D1/D3, 1 d/3 d postsurgery

To know the alterations of Ctsk in inflamed^28,29^ ODs, we first conducted IHC staining. Data showed that the level of Ctsk was notably upregulated in inflamed ODs at 3 d postsurgery (Fig. 2a). In vitro TNF-α treatment also increased Ctsk expressions both in A11 and primary OD cells (Figs. 2b and S1). And the ODs-derived Ctsk expression positively linked to the TNF-α concentrations (Figs. 2b and S1). To further verify the expressions of Ctsk in inflamed ODs, we generated Ctsk-Cre; mTmG mice, in which Ctsk expressing cells and their progenies were GFP^+^ (Fig. 2c). Using Ctsk-Cre; mTmG mice we observed that in normal ODs there were rare Ctsk expressions, but at 1 d postsurgery the ODs exhibited dramatically increased Cstk expressions (Fig. 2c). Upregulations of Ctsk in inflamed ODs continued at 3 d postsurgery (Fig. 2c). Meanwhile, we also observed significantly increased GFP^+^ cells in inflamed pulp cells in Ctsk-Cre; mTmG mice (Fig. 2c). In order to verify these findings, we also conducted immunofluorescence (IF) staining using inflamed molars derived from 3.6GFP mice. Data further proved the obviously increased Ctsk expressions in inflamed ODs, and showed the upregulation of Ctsk in inflamed pulp cells (Fig. S4C, D). In vitro TNF-α stimulation further showed that Ctsk expression and secretion in primary OD cells (Fig. S1D) and A11 were continuously upregulated along with the extended treatment time with TNF-α (Fig. S4A, B).

### NF-κB signaling orchestrates TNF-α-induced Mmp expression in ODs

**Fig. 3:**
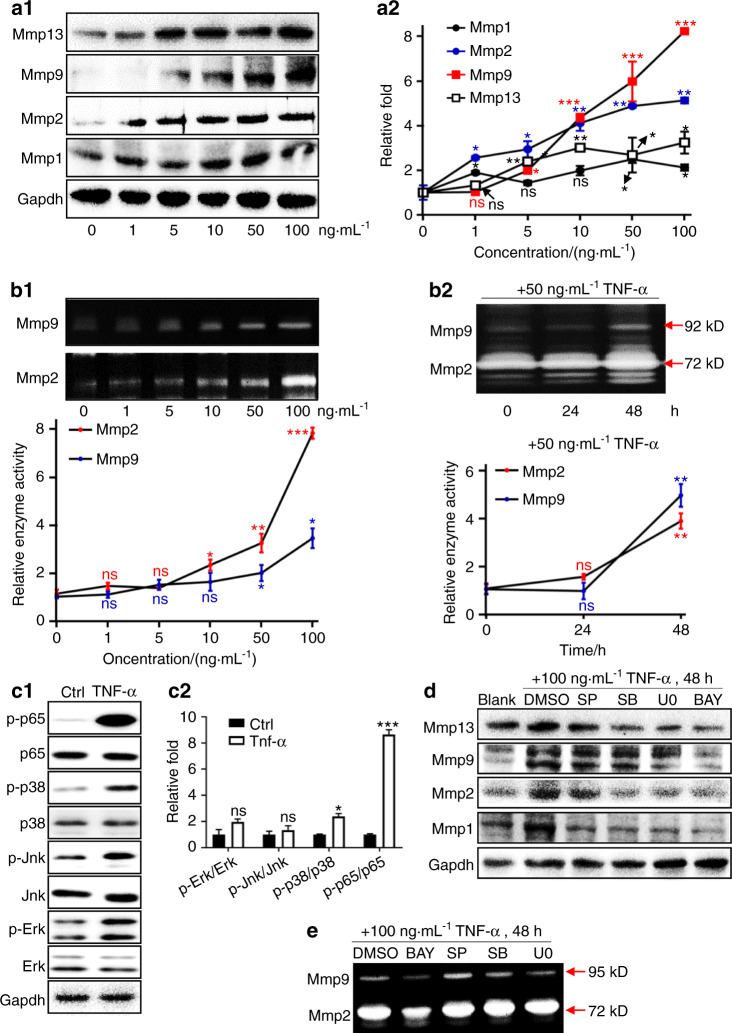
NF-κB signaling orchestrated TNF-α-induced alterations of Mmps in ODs. **a1** Representative western blot images of Mmps at 24 h post-treatments of TNF-α in A11. The concentrations listed indicated the doses of TNF-α. **a2** Quantifications of western blot, *n* = 6, and data were presented as mean and error bars of SD. **b1** Representative images and statistical analysis of zymography for Mmp2 and 9 at 24 h post-treatments of TNF-α in A11. The concentrations listed indicated the doses of TNF-α. *P < 0.05; **P < 0.01; ***P < 0.001; ns, no statistical significance. **b2** Representative images and statistical analysis of zymography for Mmp2 and 9 at different time points using 50 ng·mL^−1^ TNF-α in A11. **P < 0.01; ns no statistical significance. (**c1**) Representative western blot images of Erk, Jnk, p38, and NF-κB signaling at 30 min post-treatments of TNF-α in A11; and (**c2**) were quantifications of western blot. *n* = 3, and *P < 0.05; ***P < 0.001; ns, no statistical significance. **d** Representative western blot images of the effects of inhibiting Erk, Jnk, p38, and NF-κB signaling on rescuing TNF-α-induced Mmps in A11. **e** Representative images of zymography showing the effects of inhibiting Erk, Jnk, p38, and NF-κB signaling on rescuing TNF-α-induced matrix degradation-functions of Mmp2 and 9 in A11

To determine the underlying mechanisms of TNF-α-induced gene expression in ODs, we detected expression of key molecules in immune-regulatory pathways in dental pulp inflammation under the condition of TNF-α stimulation.^24^ First, we found that the protein levels of Mmp1, 2, 9, and 13 were positively associated with TNF-α concentrations in A11 (Fig. 3a). We found that Mmp2 and 9 were much more sensitive than Mmp1 and 13 to TNF-α in A11 (Fig. 3a2). Therefore, we next focused on the degradation activities of Mmp2 and Mmp9. Our data demonstrated that increasing TNF-α concentration significantly reinforced the matrix degradation capabilities of Mmp2 and 9 in A11 (Fig. 3b1). Meanwhile, the degradation of these two MMPs was enhanced when the treatment time of TNF-α was extended (Fig. 3b2). We further detected the main immune-regulatory pathways known in pulpitis in TNF-α-treated A11 cells.^15,24^ Our data showed that 30 mins after TNF-α treatment, the Erk, p38, and NF-κB signaling was significantly activated in A11 cells, except Jnk signaling (Fig. 3c). It is obvious that NF-κB was the most activated one among these pathways (Fig. 3c2). After confirming the blockade efficacies of specific inhibitors for these four pathways (Fig. S5C), we investigated which one was the most important signaling on mediating TNF-α-induced Mmps alteration in A11. Data showed that Jnk signaling inhibition did not obviously rescue TNF-α-induced Mmp2, 9, and 13 expressions, while the inhibitions of Erk and p38 weakened TNF-α-related Mmp1, 2, and 13 but not Mmp9 (Fig. 3d). Notably, when NF-κB was blocked, TNF-α-induced Mmp1, 2, 9, and 13 were almost rescued to as low as the levels of no-TNF-α-treated conditions (Fig. 3d). These data clearly showed that NF-κB is the most crucial signaling in orchestrating TNF-α-induced Mmps expressions in ODs. In addition, results of zymography consistently supported the core role of NF-κB in triggering TNF-α-induced Mmp2 and 9 degradation functions in A11 (Fig. 3e). Specifically, it was BAY (NF-κB inhibitor) rather than Jnk inhibitor (SP), p38 inhibitor (SB), or Erk inhibitor (U0) that significantly rescued TNF-α-induced matrix degradative capabilities of Mmp2 and 9 in A11 (Fig. 3e). Our data in Fig. 3d showed that inhibition of Jnk reduced Mmp1 expression under condition of TNF-α treatment. Considering that TNF-α treatment did not activate Jnk (Fig. 3c), and previous studies have proved that Jnk directly controlled Mmp1 transcription in diverse species and types of cells31, 32, we proposed that under the condition of TNF-α treatment the reduction of Mmp1 via Jnk inhibition was TNF-α-independent. Briefly, it was just due to the intrinsically transcriptional control of Jnk for Mmp1. To support this hypothesis, we inhibited Jnk under basal conditions without TNF-α treatment, and consequent data proved that the transcription of Mmp1 after repressing Jnk was significantly reduced in basal condition (Fig. S5A). Besides, we noticed the reduction of TNF-α-induced Mmp2 after inhibiting Erk and p38 signaling (Fig. 3d) but no obviously weakened enzymatic activity (Figs. 3e and S5B). This result implied that the residual Mmp2 levels after inhibiting Erk and p38 was still sufficient to perform enzymatic activities. Therefore, the productions of Mmp2 that were induced by 100 ng/ml TNF-α treatment were already excess for enzymatic substrates.

### TNF-α increased Rankl/Opg ratio via NF-κB signaling

**Fig. 4:**
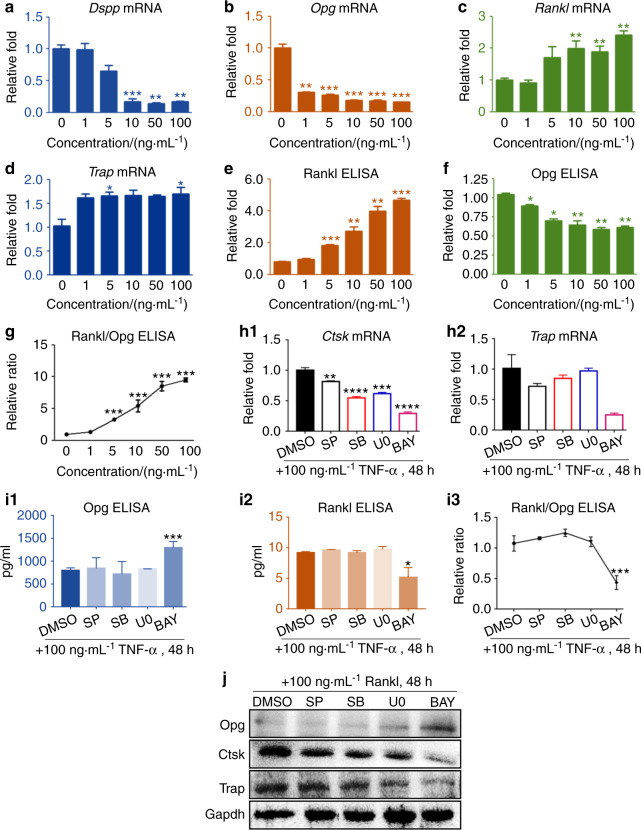
TNF-α increased Rankl/Opg ratio via NF-κB signaling. **a**–**d** RT-qPCR data of Dspp, Opg,Rankl, and Trap at 24 h post-treatments of TNF-α in A11. A two-tailed Student’s t test was used to determine the significance of difference as 0 ng·mL^−1^ TNF-α was set as control. *P < 0.05; **P < 0.01; ***P < 0.001; ns, no statistical significance. (**e**–**g**) (E&F) ELISA data of Rankl and Opg at 24 h post-treatments of 100 ng·mL^−1^ TNF-α in A11. And (G) the quantifications of secreted Rankl/Opg ratio at 24 h post-treatments of TNF-α in A11. A two-tailed Student’s t test was used to determine the significance of difference as 0 ng·mL^−1^ TNF-α was set as control. *P < 0.05; **P < 0.01; ***P < 0.001; ns, no statistical significance. **h** RT-qPCR data showing the effects of inhibiting Erk, Jnk, p38, and NF-κB signaling on rescuing TNF-α-induced (**h1**) Ctsk and (**h2**) Trap expressions in A11.A two-tailed Student’s *t* test was used to determine the significance of difference as DMSO group was set as control. *P < 0.05; **P < 0.01; ***P < 0.001; ns, no statistical significance. **i** ELISA data showing the effects of inhibiting Erk, Jnk, p38, and NF-κB signaling on rescuing TNF-α-induced (i1) Opg and (i2) Rankl autocrine-secretion levels in A11, and (i3) was the statistic results of autocrine-ratio of Rankl/Opg. A two-tailed Student’s t test was used to determine the significance of difference as DMSO group was set as control. *P < 0.05; ***P < 0.001; ns, no statistical significance. **j** Representative western blot images of the effects of inhibiting Erk, Jnk, p38, and NF-κB signaling on rescuing Rankl-induced increments of Ctsk and Trap, and meanwhile on rescuing Rankl-induced decrements of Opg in A11

To address the issues that whether ODs preferentially demonstrate deconstructive characteristics or generative functions when they respond to inflammatory stimuli, we treated A11 and primary OD cells by using TNF-α. First, our data showed that crucial resorption genes including Ctsk and Trap were continuously increased with the increment of TNF-α concentrations (Fig. S1D) and along with the time of TNF-α-treatment expanded (Fig. S4A). We found that only 3 h after TNF-α treatment, the crucial dentin-forming genes in ODs such as Dspp and Opg were dramatically reduced and the decline of these two genes was aggravated along with the increment of TNF-α concentrations (Figs. 4a, b and S1D). On the contrary, TNF-α activated expression of deconstruction genes, such as Ctsk (Figs. 2 and S1D, S4A), Rankl (Figs. 4c and S1D) and Trap (Figs. 4d and S1D, S4A) in ODs. Taken together, these results verified that ODs preferentially demonstrated deconstructive functions and impaired dentin-forming capabilities in response to TNF-α stimulation. Furthermore, we noticed that TNF-α dramatically increased the ratio of autocrine-derived Rankl versus Opg (Fig. 4e–g). These data further confirmed that A11 preferentially demonstrates catabolic functions rather than anabolic functions in response to TNF-α. This was the first time to report that TNF-α could significantly increase Rankl/Opg ratio in ODs, thus we next investigated the underlying mechanisms. We found that as an important downstream gene of Rankl, the TNF-α-induced Ctsk expressions were rescued by BAY, SP, SB, and U0, among which BAY showed the most significant efficacy (Fig. 4h1, Fig. S5D). For another vital downstream gene of Rankl signaling, TNF-α-mediated Trap increment was only rescued via inhibiting NF-κB signaling using BAY (Fig. 4h2). For TNF-α-induced Opg-reduction and Rankl-increment, only inhibiting NF-κB, but not Erk, Jnk, nor p38 signaling, rescued these events (Fig. 4i, Fig. S5D).

### TNF-α enhanced Rankl-related resorption functions via NF-κB signaling

**Fig. 5:**
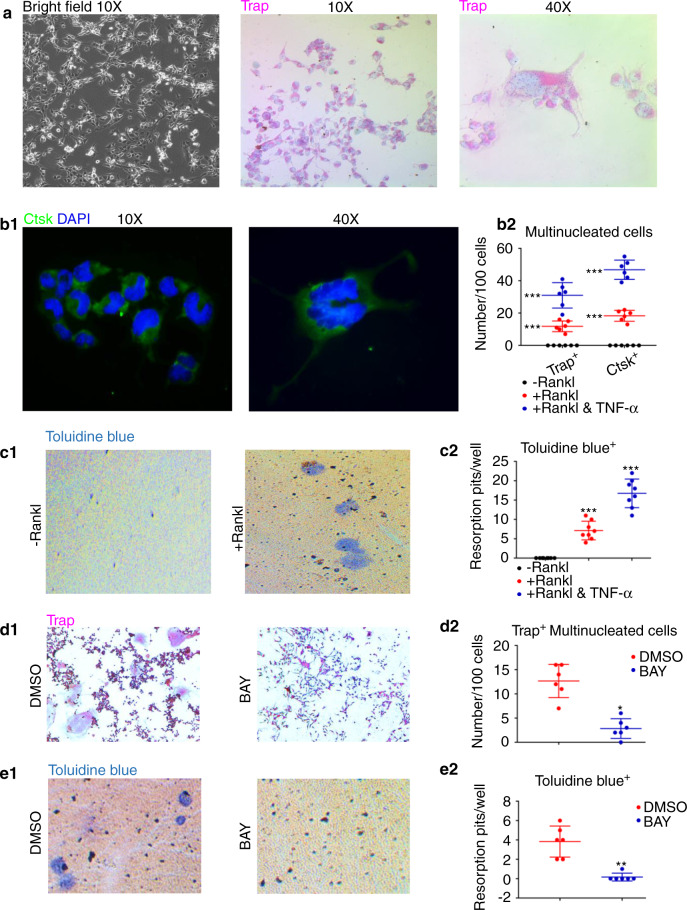
Rankl activated resorption functions of ODs via NF-κB signaling and TNF-α enhanced Rankl-related resorption functions. **a** Representative microscopic images of Rankl-induced multinucleated A11 cells. The left image was obtained in bright filed, and the middle and right ones were Trap staining results of these Rankl-treated multinucleated A11 cells, with counterstained using hematoxylin. **b** (**b1**) Representative immunofluorescence results showing Ctsk expressions in Rankl-treated multinucleated A11 cells. Cells were counterstained using DAPI. (**b2**) Statistic results of immunofluorescence results showing the effects of Rankl and TNF-α on inducing Trap and Ctsk positive multinucleated A11 cells. A two-tailed Student’s t test was used to determine the significance of difference as “-Rankl” group was set as control. ***P < 0.001; ns, no statistical significance. (**c1**) Representative images of toluidine-blue labelled resorption pits, and (**c2**) the statistic results showing effects of Rankl and TNF-α on inducing resorption functions of multinucleated A11 cells. A two-tailed Student’s *t* test was used to determine the significance of difference as “-Rankl” group was set as control. ***P < 0.001. (**d1**) Representative images of Trap staining showing the effect of inhibiting NF-κB signaling on rescuing Rankl-induced generations of Trap^+^ multinucleated cells from A11 cells, and (**d2**) relative statistic results. A two-tailed Student’s *t* test was used to determine the significance of difference as DMSO group was set as control. *P < 0.05; ns, no statistical significance. (**e1**) Representative images of toluidine-blue labelled resorption pits showing the effect of inhibiting NF-κB signaling on rescuing Rankl-induced generations of Trap^+^ multinucleated cells from A11 cells, and (**e2**) relative statistic results. A two-tailed Student’s *t* test was used to determine the significance of difference as DMSO group was set as control. **P < 0.01

Rankl/Opg ratio is crucial for hard-tissue remodeling, especially in manipulating tissue resorption.^30^ Besides, a previous study also showed that Rankl treatment could induce ODs-like cell line MDPC-23 to form Trap^+^ multinucleated cells, which owned dentin-resorption capabilities.^6^ Our findings in Fig. 4 also demonstrated that TNF-α significantly increased Rankl/Opg ratio in A11, thus we further investigated whether TNF-α-upregulated Rankl/Opg ratio could modulate ODs-related resorption functions. Intriguingly, we uncovered that inhibiting signaling of NF-κB, rather than Erk, Jnk, or p38, strongly undermined Rankl-dependent Ctsk and Trap expressions (Fig. 4j). It is interesting that inhibiting NF-κB even rescued Rankl-induced Opg decrement (Fig. 4j). We next directly detected the roles of NF-κB signaling in Rankl-dependent resorption functions. In consistent with MDPC-23,^6^ Rankl could also induce A11 cells to become Trap^+^ multinucleated cells (Fig. 5a). These multinucleated cells exerted high expressions of Ctsk (Fig. 5b1). It was important that no Trap^+^ or Ctsk^+^ multinucleated cells appeared without the Rankl treatment, and the numbers of Rankl-induced Trap^+^ and Ctsk^+^ multinucleated cells were significantly increased after TNF-α stimulation (Fig. 5b2). Besides, these A11-derived Trap^+^ or Ctsk^+^ multinucleated cells showed functional collagen resorption capacities (Fig. 5c1). The resorption functions of A11-derived multinucleated cells were remarkably enhanced via TNF-α (Fig. 5c2). Next, we proved that inhibiting NF-κB signaling significantly rescued Rankl-mediated generations of Trap^+^ multinucleated cells (Fig. 5d) and resorption functions in A11 (Fig. 5e).

**Fig. 6:**
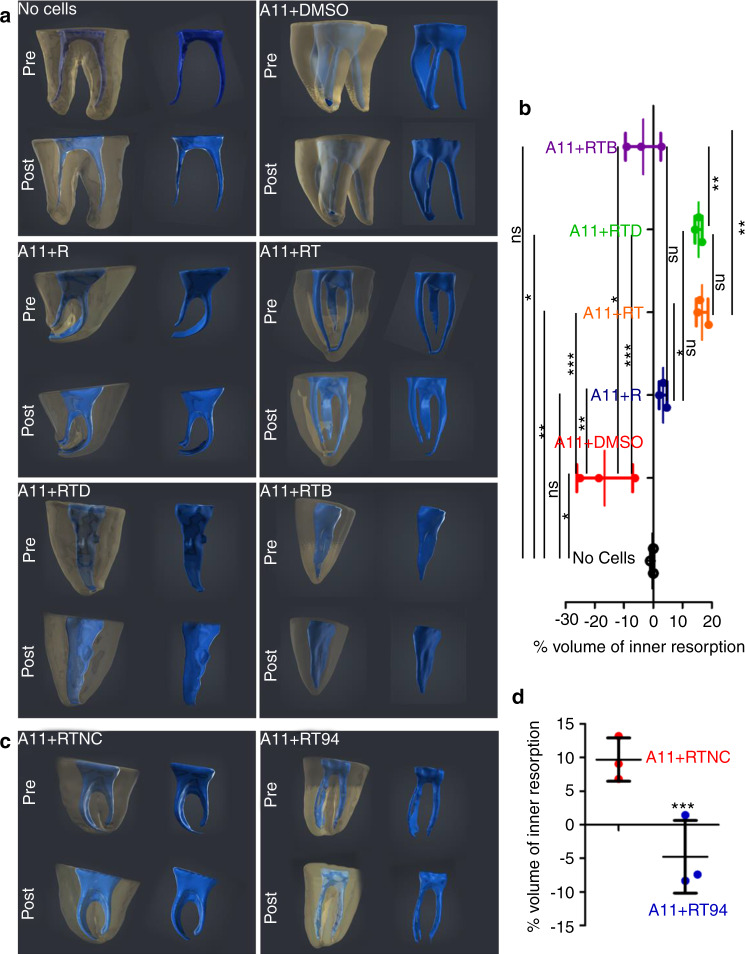
In vivo evidences indicated that the repression of NF-κB signaling and Mmps in ODs attenuated TNF-α-mediated human dental IR. (**a, c**) Representative 3D-reconstruction of μCT data. The transparent-yellow color shows the stereoscopic structures of crown-free human tooth’s hard tissue, and the blue color shows the stereoscopic structures of tooth’s internal cavity. The % volume of inner resorption was calculated as (“Postinternal cavity volume”-“Preinternal cavity volume”)/ “Preinternal cavity volume”. “Pre”, μCT data before subcutaneous transplantation; “Post”, 21d-post subcutaneous transplantation. No cells, only cell-free absorbable gelatin sponges were transplanted into human dental pulp cavity; R, Rankl treatment; RT:, dual treatments using Rankl plus TNF-α; RTD, Triple treatments using Rankl, TNF-α, and DMSO; RTB, triple treatments using Rankl, TNF-α, and NF-κB signaling-specific inhibitor BAY; RTNC, triple treatments using Rankl, TNF-α, and the solution (DMSO) of BB-94; RT94, triple treatments using Rankl, TNF-α, and the Mmps-specific inhibitor BB-94. (b&d) The statistic result of μCT data. *P < 0.05; **P < 0.01; ***P < 0.001; ns, no statistical significance

**Fig. 7:**
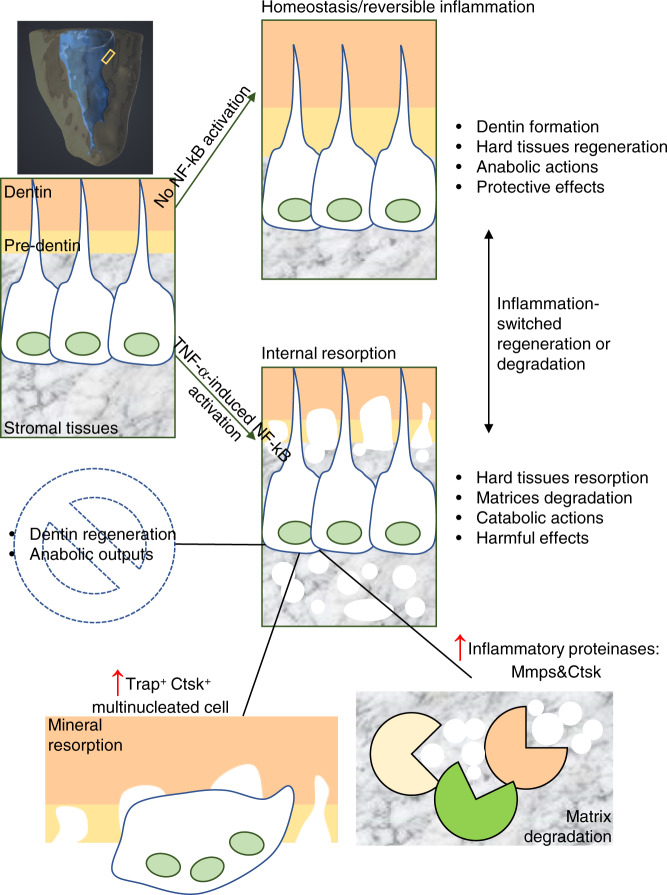
The graphical summary of findings in this study. The graphical summary of our main findings about how aberrant NF-κB signaling transforms regenerative ODs into inflammatory catabolic cell populations

Finally, we utilized an in vivo model to verify our previous findings of potential roles of inflamed ODs in IR-associated deconstruction, as well as to confirm the core effect of NF-κB signaling on orchestrating this pathological process and directly validate the importance of Mmps in this case (Fig. S5E). In brief, we found that the transplantation of normal A11 cells can help mineralization formation within decellular human tooth (Fig. 6a, b). Furthermore, we uncovered that Rankl treatment impaired hard tissue formation capability of A11 cells, but it did not cause IR-associated dentin resorption (Fig. 6a, b). Notably, when cells were dually stimulated with TNF-α and Rankl, they exhibited obvious resorption abilities (Fig. 6a, b). TNF-α and Rankl-mediated IR was proved to be efficiently abolished when NF-κB signaling or Mmps expressions were blocked via utilizing BAY11-7082 or BB-94 (the specific small molecule inhibitors respectively for NF-κB signaling and Mmps expressions) (Fig. 6a–d). Taken together, our data demonstrated that Nfκ-b signaling orchestrated IR-associated inflammatory deconstruction via enhancing proteinase functions in ODs. Aberrant NF-κB activation transformed dentin-regenerative ODs into inflammatory matrix degradation and mineral resorption populations (Fig. 7).

## Discussion

IR is an inflammatory pathological process, exerting matrix degradation and dentin resorption within the dentin-pulp complex.^1^ Previous studies mainly proposed that OCs and ONCs could mediate IR-associated tissue resorption,^1–3^ however, in this study we reported that another unexpected population, odontogenic ODs, demonstrated potential roles in IR-associated tissue deconstruction. Our data for the first time show that ODs can play crucial roles in matrix degradation and tissue resorption under inflammatory conditions.

We reported that TNF-α-induced NF-κB signaling obviously increased Rankl/Opg ratio in A11 cells, which in turn promoted Rankl-dependent formation and resorption capabilities of A11-dervied Trap^+^/Ctsk^+^ multinucleated cells. Rankl mediates the generation and resorptive functions of A11-dervied Trap^+^/Ctsk^+^ multinucleated cells via activating NF-κB signaling as well. Despite that our data and a previous study both reported that in vitro Rankl treatment could induce ODs cell lines to become Trap^+^ multinucleated cells which own dentin and collagen resorptive capabilities, in vivo clinical evidences whether this kind of ODs-derived cell exists in pulpitis tissues are totally lacking.^6^ Furthermore, as ODs are traditionally regarded as terminal-differentiated cells owning only hard-tissue forming capabilities, underlying mechanisms on how does this type of cells trans-differentiate into another typically resorptive terminal-differentiated cells deserve more future studies.^7^

In A11 cells, TNF-α preferentially promotes destructive functions and impairs dentin-forming capabilities, which is totally different from the responses of dental pulp cells.^24^ Considering the significant diversities of transcriptome, proteome, and oral pathogens-induced chemokines and cytokines of ODs and dental pulp cells, it is assumed that ODs and dental pulp cells may play different roles in inflammation regulations within dentin-pulp complex.^11,25,26^ A study has indicated that dental pulp cells may attenuate dentin resorption rather than perform deconstructive functions.^3^ Taken this study and our findings together, it implies the distinct roles of dental pulp cells and ODs in inflammation.^3^ Namely, it is assumed that during IR progression inflamed-dental pulp cells may play constructive functions, contrarily, inflamed-ODs wield deconstructive activities.

Our data demonstrate that TNF-α induces NF-κB signaling in ODs to facilitate expression and protease function of Mmps, Ctsk, and Rankl. On the contrary, TNF-α impairs Opg and Dspp expressions. These findings provide unexpected roles of ODs under inflammation conditions. As for the specific mechanisms on how NF-κB signaling manipulating these genes, more future studies are needed. Until now, it was unclear whether the in vitro dose of TNF-α used in this study had any relationship to in vivo conditions, therefore it should be noted that this is an in vitro study with limited physiological correlation.

In closing, our study for the first time demonstrates the crucial roles of NF-κB signaling in orchestrating inflamed-ODs-dependent matrix degradation and tissue resorption. These findings present novel insights into underlying mechanism of inflammation-related IR progression, and reveal the indispensable effects of NF-κB signaling on mediating inflammatory proteinases functions.

## Materials and methods

### Ethics approval

All cellular experiments and animal operations were reviewed and approved by Ethical Committees of West China School of Stomatology, Sichuan University (WCHSIRB-D-2017-062).

### Mice and animal surgery

Ctsk-Cre mice were provided by Dr. Yuuki Imai (Institute of Molecular and Cellular Biosciences, University of Tokyo, Japan).^31^ mTmG mice were purchased from Jackson Lab (stock no: 007676, Maine, USA). The OD cell reporter mice, 3.6 kb Col1α1-GFP (3.6GFP), were provided by Prof. Jianquan Feng (Baylor College of Dentistry, Texas A&M, USA). 2m-old male C57BL/6 mice were purchased from Dossy (Chengdu Dossy Experimental Animals CO., Chengdu, China). Mice experimental pulp infection model was established in accordance to our previous studies,^15^ and the successful establishment of surgeries was presented in Fig. S1A. Sham groups were abbreviated as Ctrl, and pulp exposure groups were abbreviated as Op in this study. For subcutaneous-transplantation experiments, 6-week-old Balb/c immune-deficient nude mice were purchased from Dossy (Chengdu Dossy Experimental Animals CO., China). The schematic illustration of this transplantation model was provided in Fig. S5E and this experimental procedure was modified from our previous study.^32^ The parameters and methods of μCT assessments exactly followed our previously established protocol.^32^

### Cell culture and treatments

A11 cells were kindly provided by Prof. Jianquan Feng (Baylor College of Dentistry, Texas A&M, USA).^27^ The primary GFP + OD cells of 3.6GFP mice were obtained via fluorescence-activated cytometry (FAC)-based live cell sorting. Fresh molar pulp tissues from 2m-old male 3.6GFP mice were digested using 2 mg·mL^−1^ Collagenase (Sigma, USA) for 40 min at 37 °C. Then, cells were collected and sorted via FAC to get the primary GFP + OD cells. Both A11 and primary GFP + OD cells were cultured using α-modified essential medium (α-MEM; Hyclone, UT, USA), supplemented with 10% fetal bovine serum (FBS) and 1% penicillin-streptomycin (Gibco, CA, USA) in a 5% CO_2_ and 95% air incubator at 37 °C.

### Chemical inhibitors and cytokines treatments

To efficiently block Erk, Jnk, p38, and NF-κB signaling, we respectively used their specific chemical inhibitors as follows: U0126 (U0) for Erk (10 μmol·L^−1^), SP600125 (SP) for Jnk (10 μmol·L^−1^), SB203580 (SB) for p38 (20 μmol·L^−1^) and BAY11-7082 (BAY) for NF-κB (1 μmol·L^−1^) (CST, USA).^15^ The Mmps-specific inhibitor BB-94 was purchased from Selleck (#S7155, Selleck Chemicals, USA). Cells at the confluence of 80% were pretreated with chemical inhibitors in a serum-free medium for 1 h, and then the medium was replaced with a fresh one containing 1% FBS. Efficacies of chemical inhibitors in A11 cells were verified (Fig. S5C). TNF-α (R&D, USA), Rankl (Peprotech, USA), and Mcsf (Peprotech, USA) treatments were conducted after inhibitors incubation. The concentrations and durations of TNF-α treatment were detailedly presented in relative result images.

### Histological staining

Paraffin-embedding and sectioning methods were used. After de-waxing and rehydration, slides were subjected to H&E staining or further detections. For immunohistochemistry (IHC), after rehydrated slides were further subjected to antigen retrieval for 30 min at 95 °C. Then slides were subsequently incubated overnight at 4 °C with specific first antibodies, or with IgG as negative control (Fig. S1B). At the second day, slides were washed in fresh PBS three times and then were incubated using specific second antibodies from HRP-DAB Kit (R&D System). Hematoxylin was used for counterstaining. For immunofluorescence of GFP, DAPI was incubated after the second antibody for 2 h at room temperature for counterstaining. All slides were mounted using antifluorescence quenching mounting medium, and detected. With respect to IF, nondecalcified molars were processed to become frozen sectioning slides in according to our previously established method.^32^ Then, slides were subjected to IF staining.

### Cell staining and resorption assay

To induce the Trap^+^-multinucleated resorption cells from A11, cells were cultured with 100 ng·mL^−1^ Rankl and 50 ng·mL^−1^ Mcsf (Peprotech, USA). To conduct Trap^+^ staining, and immunofluorescence of Ctsk, A11 cells were treated with Rankl and Mcsf for 7 d. For Trap staining, Trap reagents were purchased from Sigma (Trap, Aldrich Sigma, USA), and cells were fixed using 4% paraformaldehyde/PBS for 10 min and then were treated using Trap staining solution. For immunofluorescence, cells were fixed in 4% PFA for 10 min and then were treated with 0.1% Triton X-100/PBS (PBST) for 15 min. After blocking using 5%BSA in PBST for 20 min, cells were incubated with first antibodies overnight. At the second day, cells were washed with fresh PBS for three times and then were incubated using second antibody and DAPI for 2 h at room temperature. Finally, cells were washed, mounted, and detected.

The resorption assay was conducted in accordance to our previous study.^33^ Briefly, A11 cells were plated on a collagen resorption analysis chamber (Osteo Assay Surface 96-well plate, Corning, USA) at 7 d after Rankl and Mcsf induction. After cell fixation using 4% PFA chambers were subjected to be stained with toluidine-blue solution. And toluidine-blue^+^ resorption pits per chamber were counted and statistical analyzed.

### Western blot, RT-qPCR, and enzyme-linked immunosorbent assay (ELISA)

Western blot and RT-qPCR were performed using standard protocols. Briefly, for western blot cells were harvested with lysis buffer of Beyotime (China) containing protease inhibitor cocktail (SAB). The cell lysates were centrifuged at 14,000 g for 15 min at 4 °C and the supernatants were used for western blot. For RT-qPCR, total RNA was extracted using the TRIzolTM (Invitrogen) according to the manufacturer’s protocol. Complementary DNA was synthesized by using the HiScript III RT SuperMix for qPCR (Vazyme) in accordance to user manuals. Then RT-qPCR was performed in triplicate using AceQ Universal SYBR qPCR Master Mix (Vazyme) for PCR reactions on an iCycler Real-Time Detection System (BioRad, USA). The relative amount of mRNA was normalized to house-keeping gene Glyceraldehyde-3-phosphate dehydrogenase (Gapdh). ELISA detections were conducted in accordance to manual guidelines using commercial kits. Antibodies, RT-qPCR primers, and all commercial ELISA kits used in this study were listed in Table S1.

### Zymography

The zymogram was produced using a zymogram electrophoresis analysis kit (Pulilai, Beijing, China) according to manuals, which was detailedly described in our previous study.^15^ Enzymatic activities were visualized by staining with Coomassie Blue R-250 (Thermo Fisher Scientific, USA).

### Statistical analysis

Results are presented as means ± standard deviation (SD) of at least three independent biological experiments. Significance was determined using a two-tailed Student’s *t* test and the controls were respectively presented in the legends of according panels. The difference was considered as statistically significant when *P* < 0.05. All error bars shown are the calculated SD across triplicate independent experiments.

## Supplementary information

Supplemental figures

Table S1

Supplemental figure legends

## Data Availability

All data are contained within the manuscript.
