# Supplementary material for: Aberrant NF-κB activation in odontoblasts orchestrates inflammatory matrix degradation and mineral resorption
Source: Int J Oral Sci. 2022 Jan 26;14:6. doi: 10.1038/s41368-022-00159-3 (PMC8791990; doi:10.1038/s41368-022-00159-3)
Supplement: Supplementary file 1 — Supplemental figures [file 41368_2022_159_MOESM1_ESM.pdf]

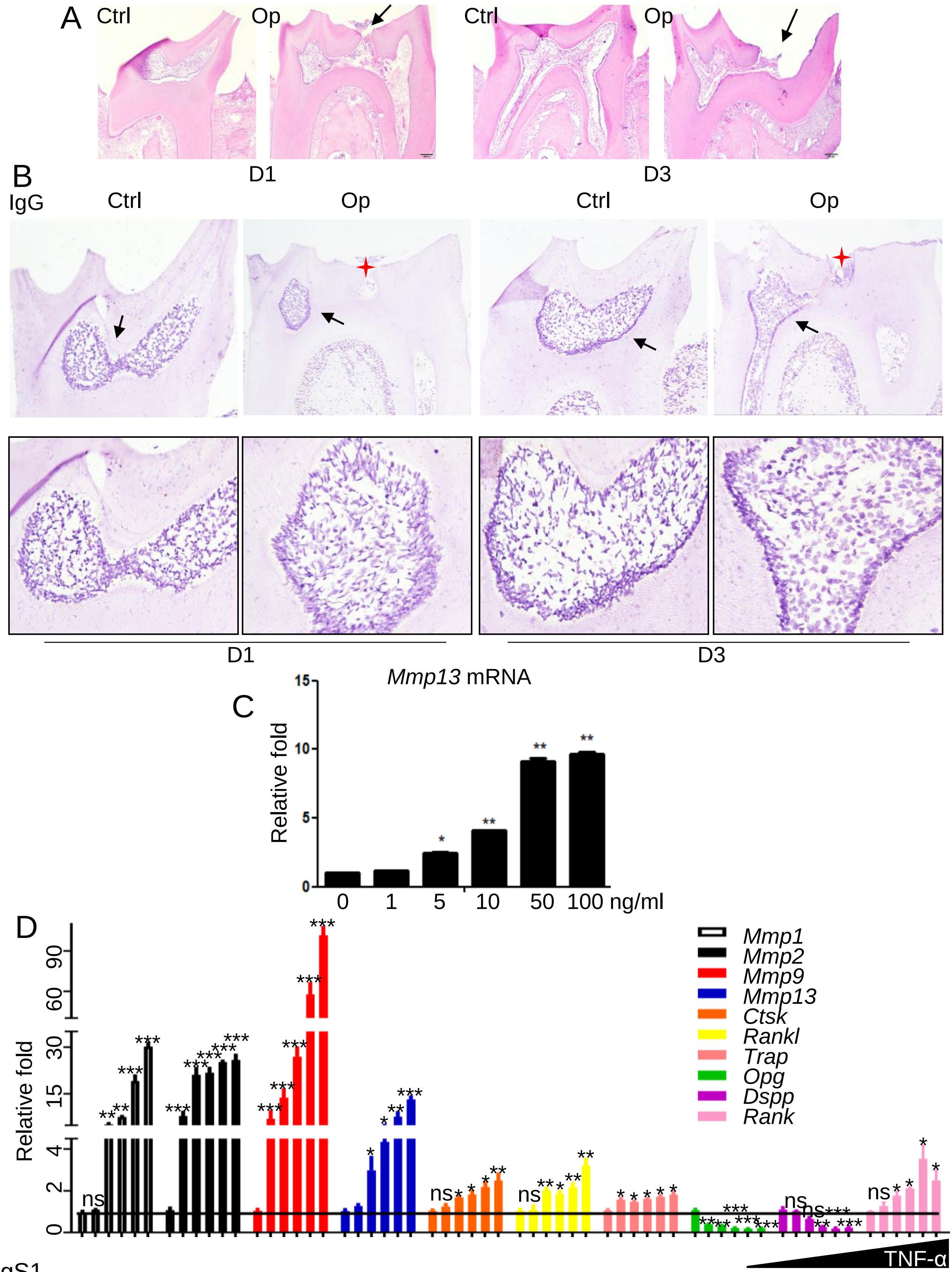
A
Ctrl
Op
Ctrl
Op
D1
D3
B
IgG
Ctrl
Op
Ctrl
Op
D1
D3
C
Mmp13 mRNA
Relative fold
0 1 5 10 50 100 ng/ml
D
Relative fold
Mmp1
Mmp2
Mmp9
Mmp13
Ctsk
Rankl
Trap
Opg
Dspp
Rank
TNF-α

FigS1

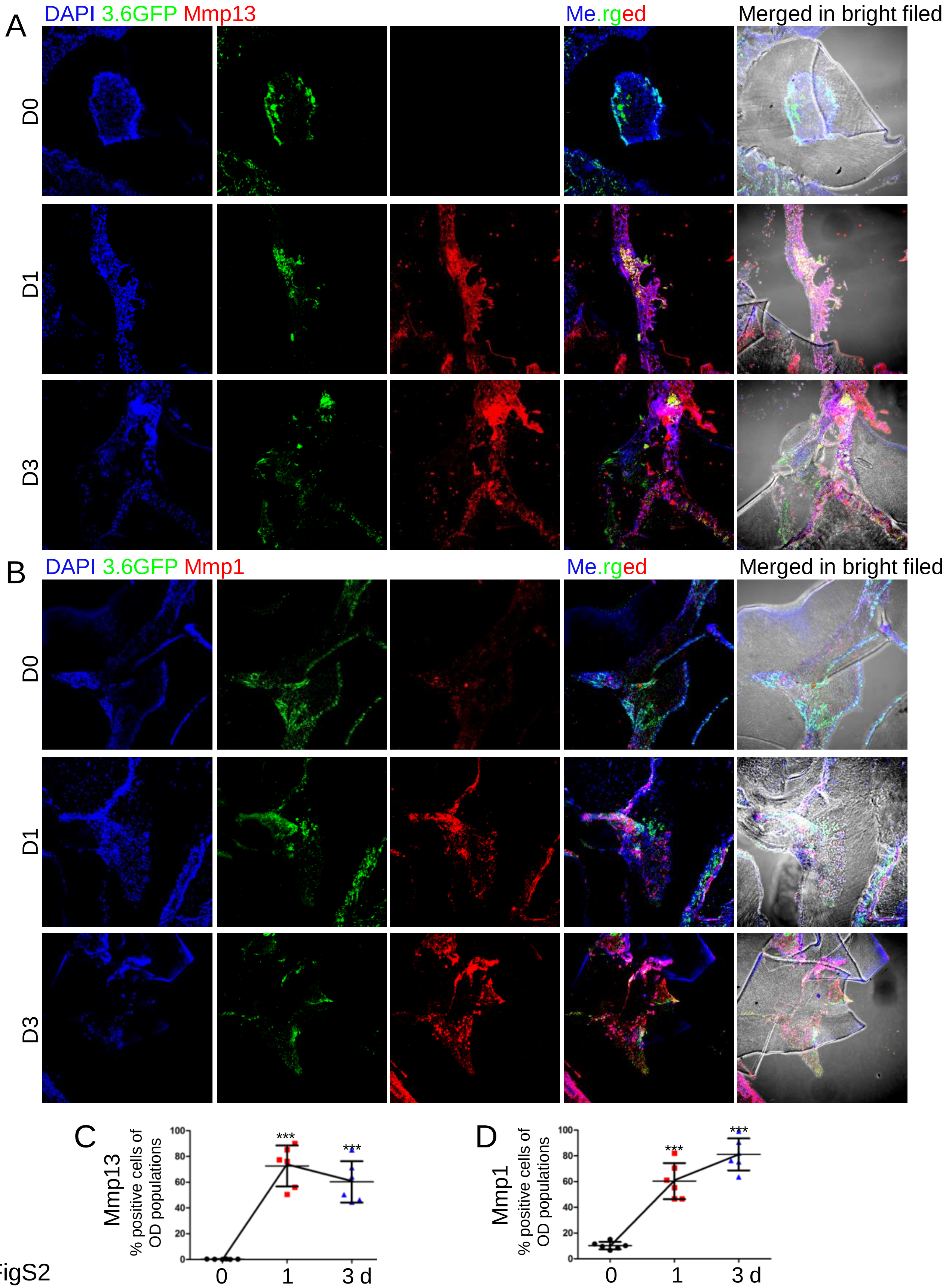
A
DAPI 3.6GFP Mmp13
Me.rged
Merged in bright filed
D0
D1
D3
B
DAPI 3.6GFP Mmp1
Me.rged
Merged in bright filed
D0
D1
D3
C
Mmp13
% positive cells of OD populations
***
***
0
1
3 d
D
Mmp1
% positive cells of OD populations
***
***
0
1
3 d

FigS2

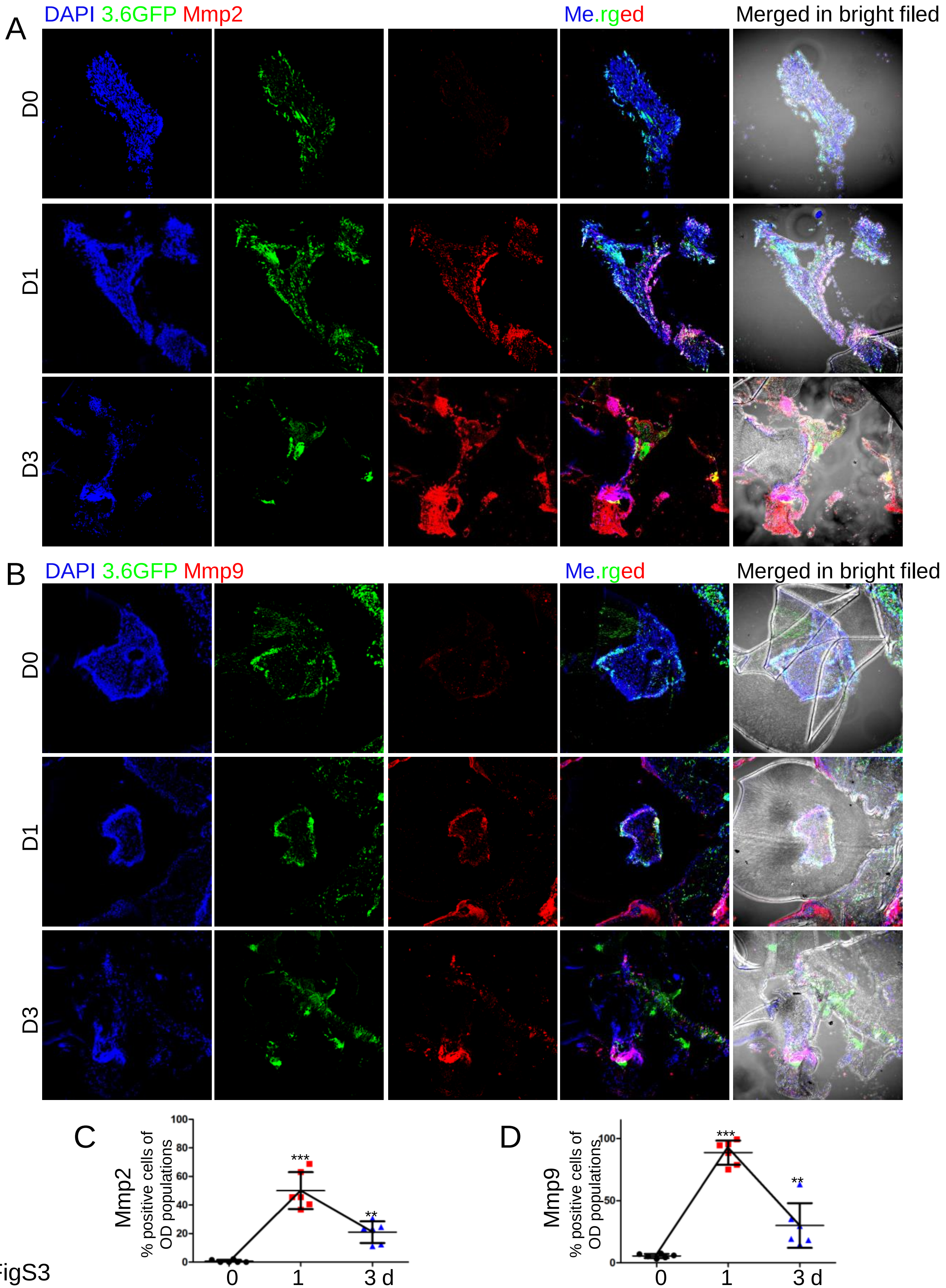
A
DAPI 3.6GFP Mmp2
Me.rged
Merged in bright filed
D0
D1
D3
B
DAPI 3.6GFP Mmp9
Me.rged
Merged in bright filed
D0
D1
D3
C
Mmp2
% positive cells of OD populations
100
80
60
40
20
0
***
**
0
1
3 d
D
Mmp9
% positive cells of OD populations
100
50
0
***
**
0
1
3 d

FigS3

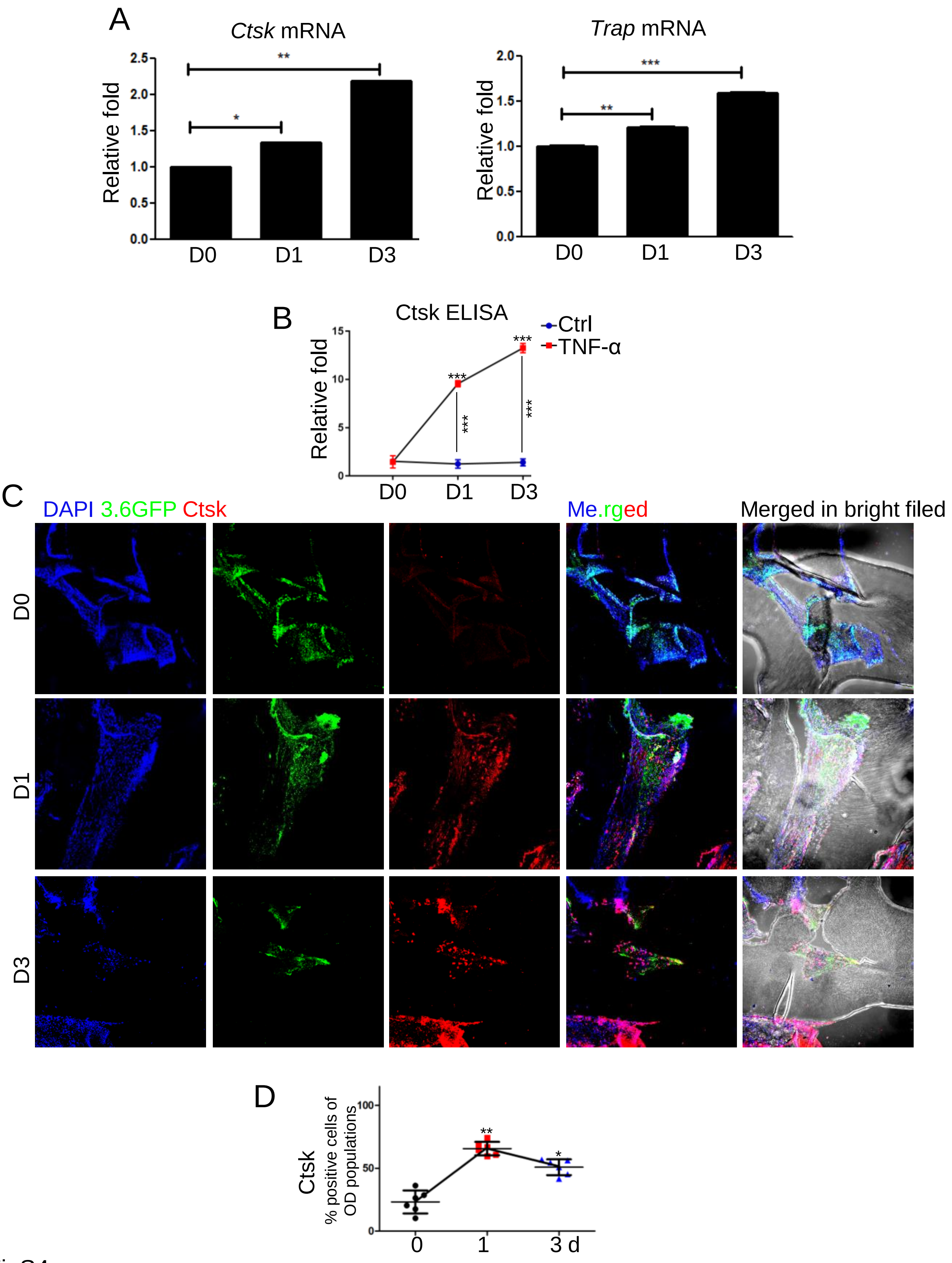
A
Ctsk mRNA
Relative fold
2.5
2.0
1.5
1.0
0.5
0.0
**
*
D0
D1
D3
Trap mRNA
Relative fold
2.0
1.5
1.0
0.5
0.0
***
**
D0
D1
D3
B
Ctsk ELISA
Ctrl
TNF-α
Relative fold
15
10
5
0
***
***
***
***
D0
D1
D3
C
DAPI 3.6GFP Ctsk
Me.rged
Merged in bright filed
D0
D1
D3
D
Ctsk
% positive cells of
OD populations
100
50
0
**
*
0
1
3 d

FigS4

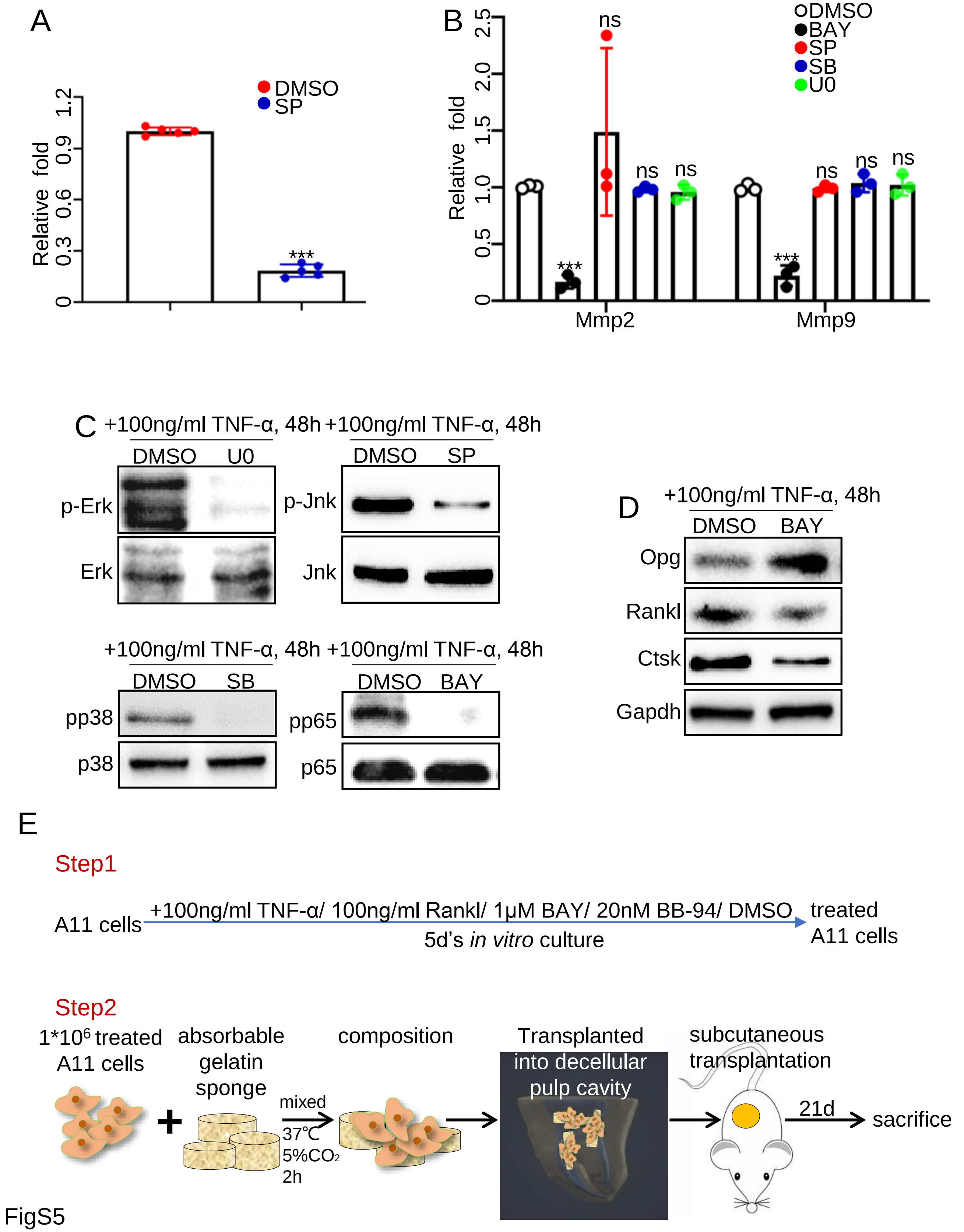

A
DMSO
SP
Relative fold
0
0.3
0.6
0.9
1.2
***
B
DMSO
BAY
SP
SB
U0
Relative fold
0
0.5
1.0
1.5
2.0
2.5
ns
***
Mmp2
Mmp9
C
+100ng/ml TNF-α, 48h
DMSO
U0
p-Erk
Erk
+100ng/ml TNF-α, 48h
DMSO
SP
p-Jnk
Jnk
+100ng/ml TNF-α, 48h
DMSO
SB
pp38
p38
+100ng/ml TNF-α, 48h
DMSO
BAY
pp65
p65
D
+100ng/ml TNF-α, 48h
DMSO
BAY
Opg
Rankl
Ctsk
Gapdh
E
Step1
A11 cells
+100ng/ml TNF-α/ 100ng/ml Rankl/ 1μM BAY/ 20nM BB-94/ DMSO
5d's in vitro culture
treated A11 cells
Step2
1*10^6 treated A11 cells
absorbable gelatin sponge
mixed
37℃
5%CO2
2h
composition
Transplanted into decellular pulp cavity
subcutaneous transplantation
21d
sacrifice

FigS5
