# Supplementary material for: Aberrant NF-κB activation in odontoblasts orchestrates inflammatory matrix degradation and mineral resorption
Source: Int J Oral Sci. 2022 Jan 26;14:6. doi: 10.1038/s41368-022-00159-3 (PMC8791990; doi:10.1038/s41368-022-00159-3)
Supplement: Supplementary file 3 — Supplemental figure legends [file 41368_2022_159_MOESM3_ESM.docx]

**Supplemental figure legends**

**FigS1. Experimental pulp infection model and detections of Mmps in inflamed ODs.**

(A) H&E staining of mice teeth after experimental pulp infection surgeries. Black arrows indicated the pulp expoure sites. Timing points listed indicate the days post-surgery. (B) Negative control of IHC using IgG to replace specific first antibodies. Black arrows indicated the areas of boxed high magnification images. Red stars indicated the sites of pulp exposure. (C) RT-qPCR data of Mmp13 at 24h post-treatments of TNF-α in A11. The concentrations listed indicated the doses of TNF-α. A two-tailed Student’s t test was used to determine the significance of difference as 0 ng·mL^-1^ TNF-α was set as control. *, *P*<0.05; **, *P* <0.01; ns, no statistical significance. (D) RT-qPCR data of FAC-sorted primary OD cell from 3.6GFP mice. The concentrations of TNF-α increase from left to right (0, 1, 5, 10, 50, 100 ng·mL^-1^). *n*=6.

**FigS2. Mmp13 and 1 increased in inflamed ODs.** (A-B) IF staining of Mmp13 and 1 in 3.6GFP mice molars. D0, 1, and 3 indicated the days post pulp exposure surgeries. (C-D) Statistics of the proportions of dual positive cells (IF positive and 3.6GFP positive) over all 3.6GFP positive OD cells. *n* =6.

**FigS3. Mmp2 and 9 increases in inflamed ODs.** (A-B) IF staining of Mmp2 and 9 in 3.6GFP mice molars. D0, 1, and 3 indicated the days post pulp exposure surgeries. (C-D) Statistics of the proportions of dual positive cells (IF positive and 3.6GFP positive) over all 3.6GFP positive OD cells. *n* =6.

**FigS4. Resorptive genes increase in inflamed ODs.** (A) RT-qPCR data of Ctsk and Trap atdifferent timing points post-treatments of TNF-α in A11. The concentration of TNF-α was 100 mg·mL^-1^ A two-tailed Student’s t test was used to determine the significance of difference as “D0” was set as control. *, *P* <0.05; **, *P* <0.01; ***, *P* <0.001; ns, no statistical significance. (B) Statistic results of ELISA showing the effect of TNF-α on Ctsk secretion. A two-tailed Student’s t test was used to determine the significance of difference as “D0” or “no TNF-α (Ctrl)” was set as control. ***, *P* <0.001; ns, no statistical significance. (C) IF staining of Ctsk in 3.6GFP mice molars. D0, 1, and 3 indicated the days post pulp exposure surgeries. (D) Statistics of the proportions of dual positive cells (IF positive and 3.6GFP positive) over all 3.6GFP positive OD cells. *n*=6.

**FigS5. NF-κB signaling induces TNF-α-mediated degradation and resorption functions in A11.** (A) RT-qPCR data of Mmp1 in A11 cells after using Jnk inhibitor without TNF-α treatment. ***, *P*<0.001. (B) The densitometry and statistical analyses of Fig 3E. ***, *P* <0.001; ns, no statistical significance. (C) Representative western blot images of the effects of inhibiting Erk, Jnk, p38, and NF-κB signaling in A11. Cell lysate were collected at 1 h post-utilizations of chemical inhibitors. (D) Representative western blot images showing the effects of inhibiting NF-κB signaling on rescuing TNF-α-mediated Opg, Rankl, and Ctsk expression alterations in A11. (E) The schematic illustration of the “human tooth subcutaneous transplantation model”, which was modified from our previous study^29^.
